# lncDIFF: a novel quasi-likelihood method for differential expression analysis of non-coding RNA

**DOI:** 10.1186/s12864-019-5926-4

**Published:** 2019-07-02

**Authors:** Qian Li, Xiaoqing Yu, Ritu Chaudhary, Robbert J. C. Slebos, Christine H. Chung, Xuefeng Wang

**Affiliations:** 1 Health Informatics Institute, University of South Florida, Tampa, FL 33612 USA; 2 Department of Biostatistics and Bioinformatics, Moffitt Cancer Center, Tampa, FL 33612 USA; 3 Department of Head and Neck-Endocrine Oncology, Moffitt Cancer Center, Tampa, FL 33612 USA

**Keywords:** lncRNA, Differential analysis, Quasi-likelihood, Head and neck squamous cell carcinomas

## Abstract

**Background:**

Long non-coding RNA (lncRNA) expression data have been increasingly used in finding diagnostic and prognostic biomarkers in cancer studies. Existing differential analysis tools for RNA sequencing do not effectively accommodate low abundant genes, as commonly observed in lncRNAs.

**Results:**

We investigated the statistical distribution of normalized counts for low expression genes in lncRNAs and mRNAs, and proposed a new tool lncDIFF based on the underlying distribution pattern to detect differentially expressed (DE) lncRNAs. lncDIFF adopts the generalized linear model with zero-inflated Exponential quasi-likelihood to estimate group effect on normalized counts, and employs the likelihood ratio test to detect differential expressed genes. The proposed method and tool are applicable to data processed with standard RNA-Seq preprocessing and normalization pipelines. Simulation results showed that lncDIFF was able to detect DE genes with more power and lower false discovery rate regardless of the data pattern, compared to DESeq2, edgeR, limma, zinbwave, DEsingle, and ShrinkBayes. In the analysis of a head and neck squamous cell carcinomas data, lncDIFF also appeared to have higher sensitivity in identifying novel lncRNA genes with relatively large fold change and prognostic value.

**Conclusions:**

lncDIFF is a powerful differential analysis tool for low abundance non-coding RNA expression data. This method is compatible with various existing RNA-Seq quantification and normalization tools. lncDIFF is implemented in an R package available at https://github.com/qianli10000/lncDIFF.

**Electronic supplementary material:**

The online version of this article (10.1186/s12864-019-5926-4) contains supplementary material, which is available to authorized users.

## Background

Long noncoding RNAs (lncRNAs) are transcripts longer than 200 nucleotides with no or limited protein-coding capability. It is estimated that, in the human genome, there are at least four times more lncRNA genes than protein-coding genes [1]. Currently, there are more than 14,000 human lncRNAs annotated in GENCODE (https://www.gencodegenes.org/). Overall, lncRNA genes have fewer exons, lower abundance and are under selective constraints compared to protein-coding genes. LncRNAs are involved in diverse regulatory mechanisms and in some critical pathways. For example, they can act as scaffolds to create higher-order protein complexes, as decoys to bind sequester transcription factors, and as guides of protein-DNA interactions [2–4]. Emerging evidence suggests that lncRNAs serve as essential regulators in cancer cell migration and invasion, as well as in other cancerous phenotypes [5, 6]. Therefore, lncRNAs are becoming attractive potential therapeutic targets and a new class of biomarkers for the cancer prognosis and diagnosis. For example, the lncRNA PCA3 (prostate cancer antigen 3) is an FDA-approved biomarker for prostate cancer prediction. The overexpression of lncRNA HOTAIR in breast cancer patients is reported to be associated with patient survival and risk of metastasis [7]. Another important lncRNA ANRIL (CDKN2-AS1) is one of the most frequently alerted genes in human cancers and has been reported to increase the risks of diverse cancers.

Although a large number of lncRNAs have been identified, only a very small proportion of them have been characterized for cellular and molecular functions. Similar to protein-coding genes, the biomarker discovery of lncRNAs can start from a genome-wide differential expression (DE) analysis. One advantage of lncRNAs research in cancer is that we can leverage the large collection of previously published RNA-seq data and perform secondary analyses. Unlike the miRNAs counterparts, the expression of a large number of lncRNAs can be detected by standard RNA-seq with sufficient sequencing depth. Through downloading RNA-seq BAM files and recalling using GENCODE genomic coordinates, more than 8000 human tumor samples across all major cancer types in The Cancer Genome Atlas (TCGA) and other published studies have been re-analyzed for the lncRNAs expression profile [8, 9]. There is a limited number of non-tumor samples sequenced for RNA-seq in TCGA. If necessary, the database such as the GTEx (http://gtexportal.org) can serve as additional tissue-specific controls, which provides over 9600 RNA-seq samples across 51 tissues.

lncRNAs expression data have several features that pose significant challenges for the data analysis, including low abundance, large number of genes, and rough annotations. To ensure detection reliability, a common practice is to filter out lncRNA genes with low average Reads Per Kilobase per Million mapped reads (RPKM), e.g. < 0.3. We recommend using the two-step filter proposed by Yan et al. [9]: first eliminates the genes with 50th-percentile RPKM =0, and then only keep the genes with 90th-percentile RPKM < 0.1. About two-thirds of lncRNAs are excluded after this filtering procedure. Interestingly, excess zeros or low expression values are still observed in the downsized dataset. It is well known that excess zero read counts in RNAseq data can distort model estimation and reduce power in differential expression analysis. The popular R packages DESeq2 and edgeR assume a negative binomial (i.e. over-dispersed Poisson) distribution for the count data. Methods based on zero-inflated negative binomial (ZINB) and zero-inflated GLM have been proposed to explicitly address the issue of excess zeros in RNA-seq data [10]. These methods have been recently applied to single-cell RNA-seq (scRNA-seq) data, which has high dropout rates. Since the difference in gene expression variance is biologically interesting, multiple methods have been developed to incorporate the testing of variance in the differential model. However, for biomarkers in clinical settings, genes with pronounced group contrast in mean expression level usually have more translation value. Gene-wise expression variability can generate from different sources and vary widely from study to study, especially with different normalization methods. Hence, we focus on the group comparison of mean gene expression levels in this study.

In a large-scale secondary analysis of expression data such as in lncRNAs studies, it is common to only have access to normalized data (such as RPKM), due to either limited data availability or less ideal performance of other normalization methods [11, 12]. Packages such as DESeq2, however, are not applicable to lncRNA normalized counts because they do not allow non-integer normalized expression or zero as input. In this case, a plausible practice is to round continuous expression values into integers and then to add 1 to each value to remove zeroes. Another commonly-adopted approach is using *log*_2_(*x* + 1) transformed normalized data in R package like limma [13], i.e., assuming a log-transformed Gaussian distribution as in microarray intensity levels. The core function in limma, which runs a moderated t-test after an empirical Bayes correction, is more generic and more suitable for the differential expression of processed lncRNA expression data. In a very recent study, a total of 25 popular methods for testing differential expression genes were comprehensively evaluated with special emphasis on low-abundance mRNAs and lncRNAs [14]. It was observed that linear modeling with empirical Bayes moderation (implemented in limma with variance stabilizing transformation [15], voom [16] or trend), and a non-parametric method based on Wilcoxon rank sum statistics (implemented in SAMSeq) showed overall good balance of false discovery rate (FDR) and reasonable detection sensitivity. However, none of the methods compared can outperform other tools and all tools exhibited substandard performance for lncRNAs in terms of differential testing, often with higher FDR and true positive rate (TPR) than for mRNAs. This study also concluded that accurate differential expression inference of lncRNAs requires more samples than that of mRNAs. Even methods like limma can exhibit an excess of false discoveries under specific scenarios, making these methods unreliable in practical applications.

In this paper, we first investigated the distribution of lncRNA and low-abundance mRNA via the relation between gene-wise coefficient of variation and mean. The patterns for these RNAs were compared with high abundant mRNA, providing evidence for an underlying Exponential distribution in most genes of lower expression, especially those in lncRNA. Based on the assumption of Exponential-distributed non-zero abundance for the majority of lncRNA genes, we presented the lncDIFF, an efficient and reliable toolset in a zero-inflated Exponential quasi-likelihood strategy on the Generalized Linear Model. The quasi-likelihood provides unbiased estimations for biological group effect on lncRNA gene expression, including a small proportion of lncRNA genes with expression following Negative Binomial or Log Normal distribution. It thus provides a simple and versatile approach to model gene expression data without making strong distributional assumptions about the underlying variation, but still being compatible with existing RNA-Seq quantification and normalization tools. The flexibility in allowing for the estimation of calibration and variance parameters is especially important for lncRNAs differential analysis. lncDIFF is thus able to integrate desirable features from the aforementioned two top-performing methods (limma and SAMSeq [14]) for lncRNA differential analysis. lncDIFF is compared with existing tools using an extensive simulation study and lncRNA DE analysis on TCGA head and neck squamous cell carcinomas (HNSC), with data downloaded from TANRIC [8]. Results suggest that lncDIFF is powerful and robust in a variety of scenarios and identifies DE lncRNA genes of low expression with higher accuracy.

## Results

### Simulation study to assess lncDIFF performance

We conducted a comprehensive simulation study to assess the performance of lncDIFF, and compared with existing common tools DESeq2, edgeR and limma (with log transformation), along with recently developed single-cell tools zinbwave [17] incorporated in DESeq2 (i.e. zinbwave+DESeq2), and DEsingle [18]. We rounded decimals to integers as input for DESeq2 and selected the quasi-likelihood estimation method in edgeR. lncDIFF and the compared methods were applied to low-abundance RNA-Seq genes sampled from zero-inflated NB or LN families. ShrinkBayes [19, 20] is a Bayesian approach that also adopts zero-inflated NB for low counts RNA-Seq DE analysis, designed for small sample size studies but slower in computation compared to other tools. Hence, ShrinkBayes was not applied to simulated datasets, and was compared to lncDIFF only based on TCGA HNSC datasets.

We adopted the gene-wise estimated dispersion or log variance from TCGA HNSC [21] lncRNA RPKM as the density parameters for data generation. Based on the dispersion and log variance estimate for the data in this TCGA study, we used *ϕ* = 1, 2, 10, 20, *σ*^2^ = 0.01, 0.25, 1, 2.25, and fixed *ϕ*, *σ*^2^ values to generate RPKM of each genes across all samples in the same simulation scenario. Each scenario was defined by the unique gene-wise nonzero proportion π = 0.5, 0.7, 0.9, sampling distribution function (NB or LN) and value of *ϕ*, *σ*^2^, with sample size varying at *N* = 100, 200, 300. In order to generate data similar to lncRNA RPKM, we first obtained binary outcomes (0–1) for all samples in one scenario from the Bernoulli sampling, and then replace the 1’s by positive abundance value sampled from NB or LN densities. The HNSC study includes 40 pairs of matched normal-tumor tissues. We used the 40 normal samples to calculate the mean RPKM as baseline group parameter *β*_*i*1_ in simulation. Similar to the common filtering criteria in existing lncRNA analysis, we removed the genes in the real data with mean RPKM < 0.3 [22, 23] and zero expression in more than half of the samples, reducing to 1100 genes used for simulation.

**Fig. 1:**
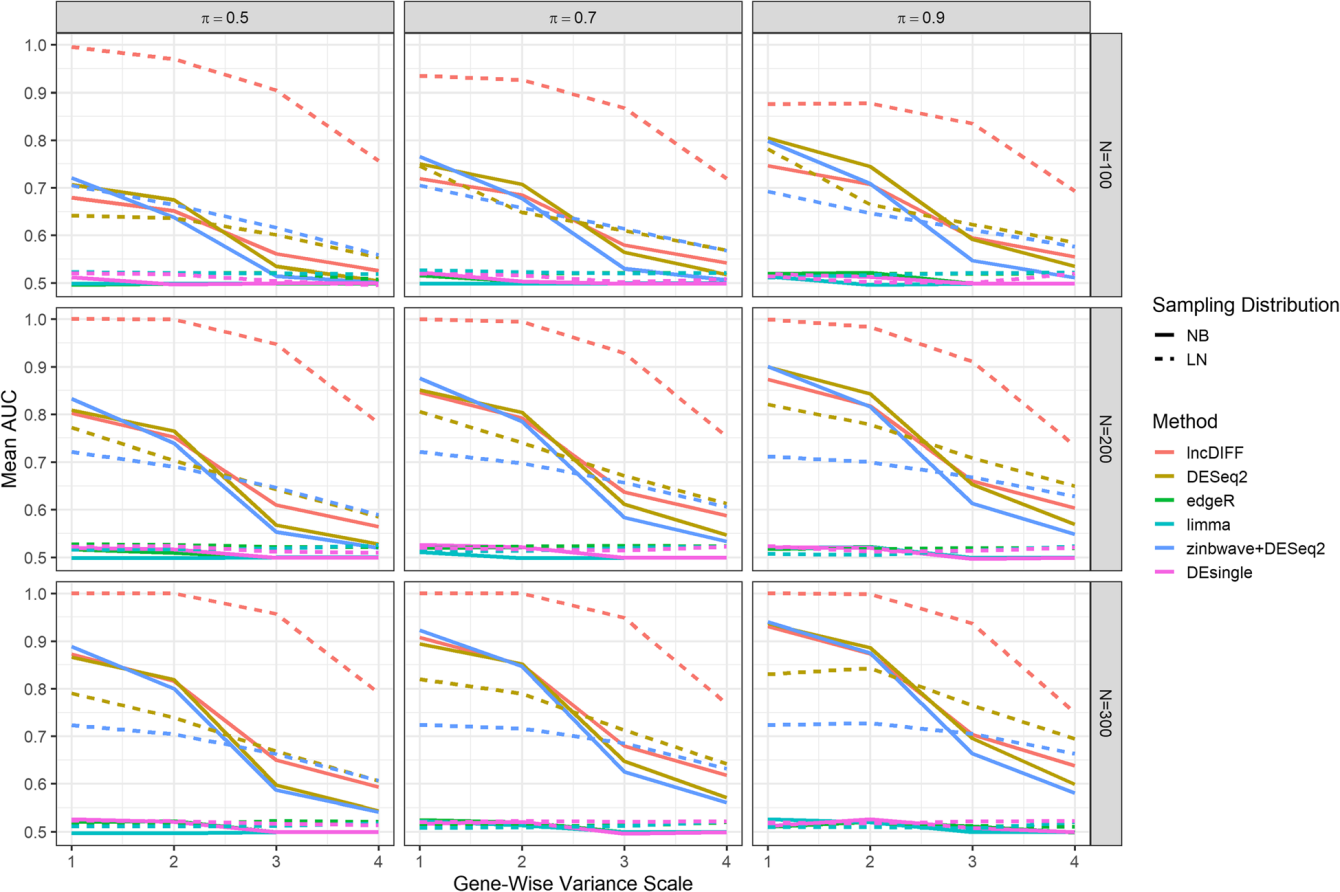
AUC of ROC curve for DE analysis on simulated data. Scenarios are in the order of true density, proportion of non-zero expression values, variance level. Labels ‘1, 2, 3, 4’ on x-axis represent gene-wise variance scales from the smallest to the largest

In the simulation study, we only considered two-group comparison to illustrate the contrast between different methods. RPKM of the first group was randomly generated by the specified density function and the baseline parameter, while the second group had the mean parameter of the baseline times a shift, i.e., the tumor/normal fold change in TCGA HNSC data. We manually set the shift between two simulated groups at 1 if the absolute log2 fold change for the corresponding gene is less than 0.5. Simulated genes with between-groups shift at 1 are the null genes and the remaining are DE genes. For each simulated scenario, we generated 100 replicates to assess the performance of different methods by the mean of false discovery rate (FDR) and true positive rate (TPR), and area under the curve (AUC) of receiver operating characteristics (ROC) with FDR threshold 0.05. We ordered the scenarios by the scale of variance (with 1–4 representing the smallest to the largest), proportion of nonzero expression, and sample size to investigate the impact of parameters on performance metrics. Figure 1 and Additional file 1: Figures S4-S5 presented the AUC, FDR and TPR of all scenarios, illustrating that lncDIFF outperforms the other methods, especially for scenarios with LN density.

AUC for all methods in Fig. 1 decrease as the gene-wise variation increases, and lncDIFF’s performance is close to the optimal method (DESeq2) for NB density. The change of AUC across different sample sizes implies that adding more samples improves the performance of lncDIFF and DESeq2, but does not have impact on edgeR, limma and DEsingle. Furthermore, the AUC of lncDIFF in NB density is equivalent to or slightly larger than that of DESeq2 at sample size *N* = 300. According to AUC and TPR, the outperformance of DESeq2 compared to lncDIFF in NB sampling was not as pronounced as the outperformance of lncDIFF compared to DESeq2 in LN distribution. The single-cell RNA-Seq tool zinbwave improves DE detection power of DESeq2, but only for small gene-wise variance and many samples having simulated counts > 4. TPR of limma was higher than the other methods except for lncDIFF on LN distributed data in smaller sample sizes. On the other hand, the FDR shows that lncDIFF has similar performance of DESeq2 in most scenarios regardless of density and greatly outperforms the other two methods, although lncDIFF in large-variance LN scenarios presents performance close to edgeR and limma. The change of performance of DESeq2 under either sampling distribution brought by zinbwave illustrates that it is the Exponential likelihood rather than zero-inflated point mass contributing towards the outperformance of lncDIFF. In summary, lncDIFF is an ideal method for DE analysis of lncRNA RPKM with different distributions, while DESeq2 is a preferred tool if the non-zero counts are relatively high and NB-distributed.

### Application of lncDIFF to TCGA HNSC data

**Fig. 2:**
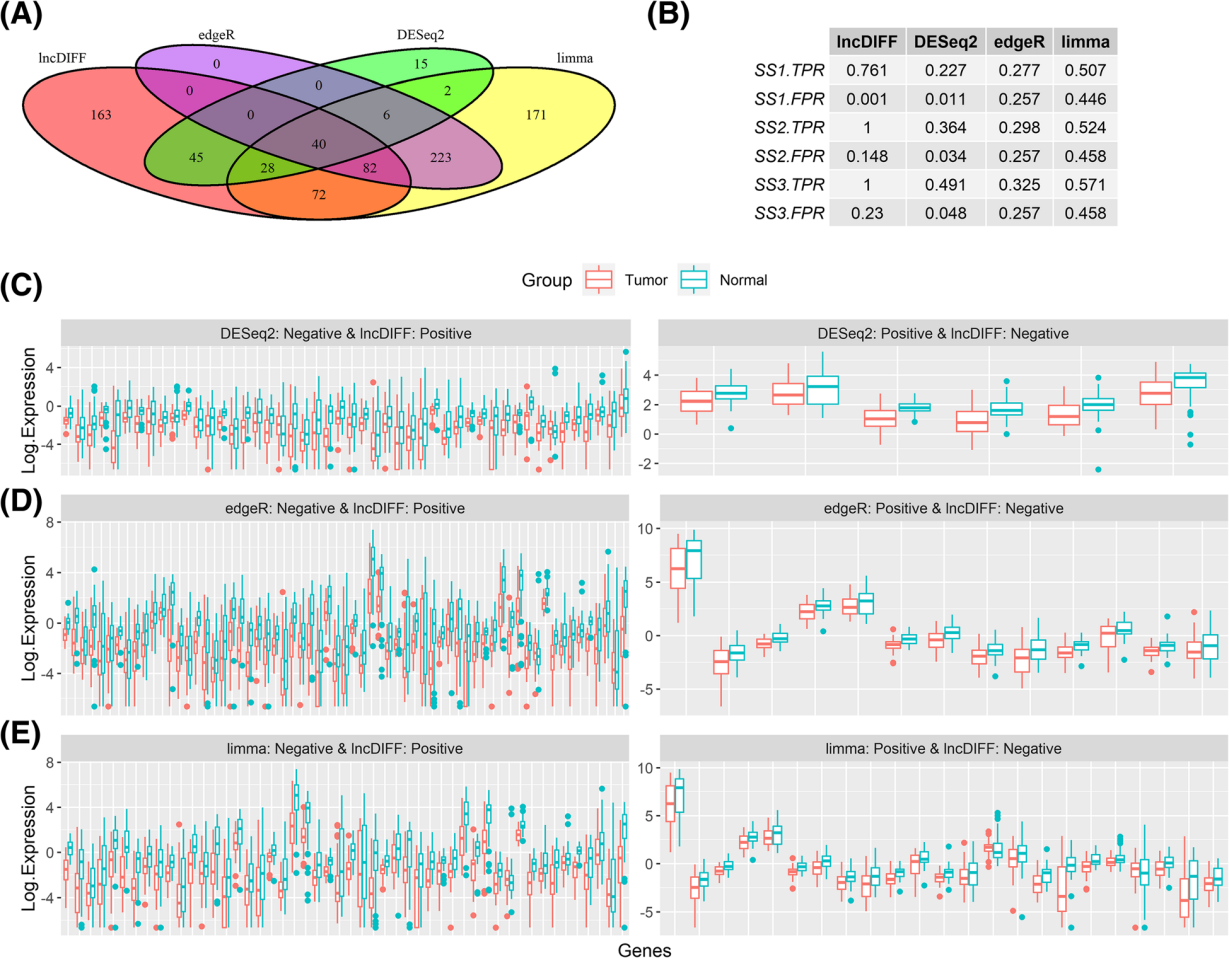
Performance of lncDIFF, DESeq2, edgeR and limma on TCGA HNSC matched tumor-normal samples. (A) Venn diagram for DE genes identified by each method. (B) TPR and FPR for each method based on surrogate DE gene sets SS1-SS3 defined by log2 fold change > 0.5, 1.0, 1.5. (C)-(E) Boxplots for tumor vs. normal log2 RPKM of genes detected as positive by one method and negative by another

**Fig. 3:**
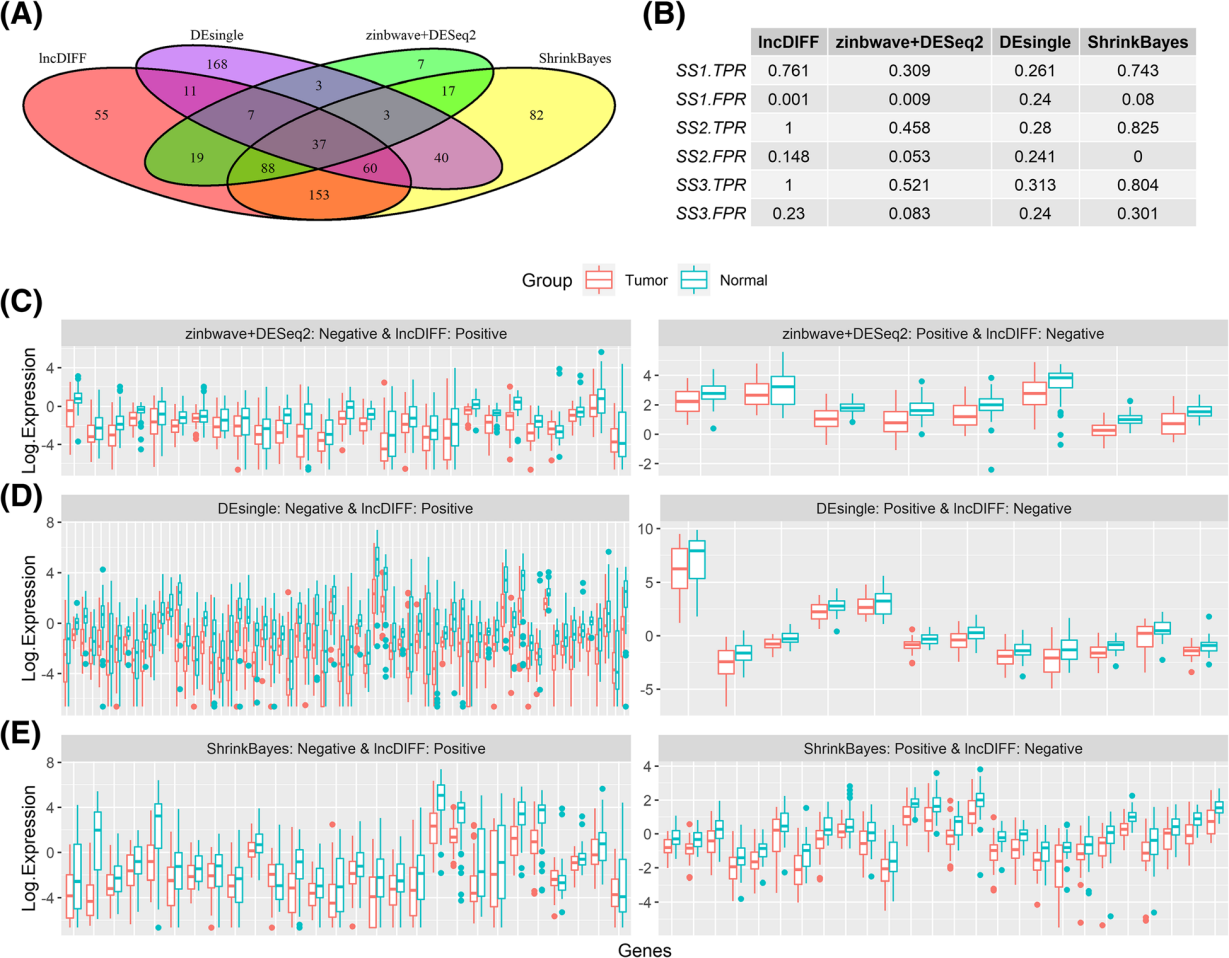
Performance of lncDIFF, zinbwave+DESeq2, DEsingle and ShrinkBayes on TCGA HNSC matched tumor-normal samples. (A) Venn diagram for DE genes identified by each method. (B) TPR and FPR for each method based on surrogate DE gene sets SS1-SS3 defined by log2 fold change > 0.5, 1.0, 1.5. (C)-(E) Boxplots for tumor vs. normal log2 RPKM of genes detected as positive by one method and negative by another

We employed the above methods along with ShrinkBayes to perform DE analysis on the TCGA HNSC lncRNA data for matched (or paired) tumor and normal samples, with results summarized in Figs. 2-3. The Venn diagrams in Figs. 2-3(a) show the overlap and difference of the DE genes identified by different methods. We do not have prior knowledge about the ‘true’ DE genes for HNSC tumor vs normal. Thus, the genes with log2 fold change > 0.5, 1, or 1.5 were considered as ‘pseudo’ or ‘surrogate’ DE genes, respectively, labeled as Surrogate Set 1–3 (SS1- SS3) of DE genes. For each set, the proportion detected by each method is a surrogate true positive rate (SS1.TPR-SS3.TPR), while the surrogate false positive rate (SS1.FPR-SS3.FPR) is the percentage of those not in surrogate DE genes set but detected as positive by each method, listed in Figs. 2-3(b). The significance threshold for tumor vs normal DE gene is adjusted *p*-value< 0.05. We further visualized the contrast between lncDIFF and the other methods by boxplots in Figs. 2 and 3c-e**,** with each panel showing the tumor vs normal group effect on the lncDIFF positive genes identified as negative by other methods. We only include the genes with upregulation for normal tissues and LFC > 0.5 in the boxplots.

The results in Figs. 2-3(b) suggested that lncDIFF provided ideal power or alternative TPR (75%) in DE analysis for LFC < 0.5, with approximated FPR below 5%. ShrinkBayes has detection power close to lncDIFF only for DE genes in SS1. Figures 2 and 3c-e displayed the group contrast on genes identified as DE (positive) by one method but non-DE (negative) by the other method using boxplot of RPKM at log2 scale per group. The group contrast on the DE genes identified only by lncDIFF was much larger than that in DE genes identified only by each of the compared methods. In other words, lncDIFF identifies ‘true’ DE genes with more power and is less likely to ‘miss’ the DE genes with pronounced group contrast.

We also applied the same analysis to the unpaired tumor (*N* = 426) and normal (*N* = 40) samples in the TCGA HNSC study by lncDIFF, and compared the top significant genes in the paired and unpaired DE analysis results (Table 1). There are 11 overlapped genes in the top 20 significant gene list of paired and unpaired analysis, some of which are associated with overall survival time. For each the overlapped significant genes, we divided the 426 HNSC tumor samples into two groups by the median of RPKM per DE gene, and then apply Cox Proportional Hazard model to survival association analysis. The Kaplan-Meier curves and the log-rank test *p*-values reveal marginal or significant associations between genes *ERVH48–1*, *HCG22, LINC00668, LINC02582* and the overall survival months (Additional file 1: Figure S6). For the same set of HNSC tumor samples, we also used the mRNA normalized counts to select 20 mRNA genes highly correlated with the 11 tumor-normal DE lncRNA genes by Spearman correlation (Additional file 3).

## Discussion

### Computational performance of lncDIFF

The GLM group effect estimation was implemented in the R function ZIQML.fit, separated from the likelihood ratio testing included in another function ZIQML.LRT. The GLM group effect estimate in lncDIFF is based on the zero-inflated Exponential likelihood with either identity or log link function, which is also valid and unbiased for low-expression lncRNA genes distributed as NB or LN. The choice of link function does not have any impact on the group effect estimate and LRT results (Table 2), but the log link function can avoid NA values produced in numerical optimization of the likelihood function. lncDIFF provides the option of either identity or log link function in the function ZIQML.fit.

**Fig. 4:**
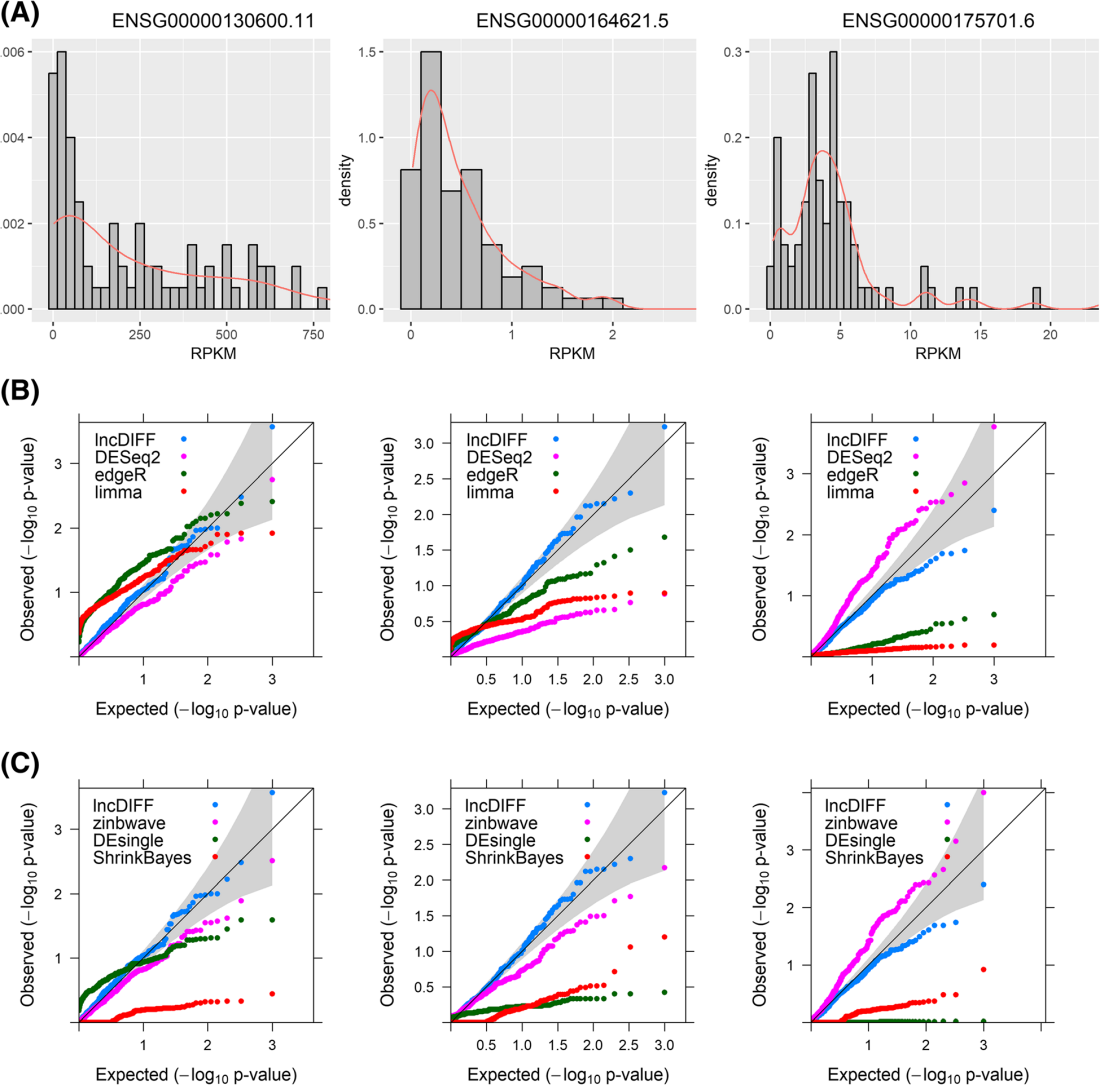
QQ plots of simulated null p-values for genes in TCGA HNSC study. (A) Histogram and density plot of RPKM for each genes. (B) Corresponding QQ plot of null p-values simulated by shuffling the samples for lncDIFF, DESeq2, edgeR and limma. (C) Corresponding QQ plot of null p-values simulated by shuffling the samples for lncDIFF, zinbwave+DESeq2, DEsingle and ShrinkBayes

The distribution of *p*-values from lncDIFF was also investigated and compared with the other methods in TCGA HNSC tumor vs. normal analysis, using simulated p-values from sample permutation. We randomly selected three genes with different RPKM density patterns to generate the null p-values and then visualized the *p*-values distribution via QQ plots in Fig. 4. Figure 4(b)-(c) showed that the p-values of lncDIFF and DESeq2 (with or without zinbwave) were close to the expected distribution aligned on the identity line, while the other methods resulted in a large proportion of small *p*-values (< 0.1). The histogram and density plot of RPKM presented in Fig. 4(a) implied that the null p-values of lncDIFF and DESeq2 for higher expressed lncRNA genes (ENSG00000130600.11) followed the expected uniform distribution, while those for low abundance genes (ENSG00000152931.7, ENSG00000153363.8) may deviate from the assumed uniform distribution. To avoid the distorted distribution of LRT *p*-values, we also implemented the option of empirical p-value and FDR based on the zero-inflated Exponential likelihood in the R function ZIQML.LRT.

We further illustrated the computation efficiency of lncDIFF by running on the TCGA HNSC matched tumor-normal samples with ~ 1130 filtered genes. The processing time (in seconds) of this biological data analysis by lncDIFF, DESeq2, edgeR, limma, zinbwave+DESeq2, DEsingle, and ShrinkBayes are 3.17, 4.31, 3.37, 0.02, 55.33, 52.67, and 341.47, respectively. If the option of simulated p-value is enabled, the running time of lncDIFF on this real dataset is increased to 267.86 s for default 100 permutations, but the correlation between observed and simulated p-values or FDR’s is around 0.9.

### lncDIFF on different normalization methods

In order to illustrate normalization methods having no impact on lncDIFF performance, we simply applied lncDIFF DE analysis to three different types of normalized counts (i.e., FPKM, TMM and UQ) of low abundance mRNA in TCGA HNSC tumor-normal samples (*N* = 546). The low abundance genes were selected with mean FPKM in the range of (0.3, 2) and no more than 20% zero expression, similar to the majority of lncRNA genes. The Pearson correlation of log10 adjusted *p*-values between the three normalization methods were FPKM vs. TMM 0.82, FPKM vs. UQ 0.92, TMM vs. UQ 0.96, implying similar DE analysis results. Therefore, we only used RPKM of lncRNA in TCGA HNSC to illustrate the application and performance of lncDIFF in this study. In addition to TMM and UQ, the quasi-likelihood parameter estimation in lncDIFF is still robust for gene expression processed from model-based RNA-Seq quantification and normalization tools, such as RSEM [24], baySeq [25], and QuasiSeq [26]. Hence, the lncDIFF DE analysis can be incorporated in existing RNA-Seq quantification and normalization pipeline, regardless of the models employed in the preprocessing tools.

## Conclusions

We implemented GLM with zero-inflated Exponential likelihood and LRT for either identity or logarithmic link function in lncDIFF, along with an option of simulated p-values and FDR generated from permutations. This package allows the input expression matrix to be either continuous or discrete and requires group or phenotype factor provided in the design matrix format. lncDIFF is a powerful differential analysis tool for zero-inflated low-counts RNA-Seq data, especially for lncRNA and large-scale studies, with improved DE detection power and computational performance compared to others. This is an efficient DE analysis method compatible with various RNA-Seq quantification and normalization tools.

## Methods

### Low-abundance RNA-Seq data distribution

In RNA-Seq analysis, the type of RNAs and the selected alignment, quantification and normalization tools usually have substantial impacts on the distribution pattern of transcript abundance [27], especially on the level of gene expression dispersion, i.e. the mean-variance relation. Most of the existing RNA-Seq analysis tools, such as DESeq [28], edgeR [29], and baySeq [25], estimate gene-wise dispersion to perform normalization or downstream differential expression analysis. However, algorithms based on gene-wise dispersion may not be suitable for low counts in RNA-Seq studies, such as lncRNA and low-expression genes in mRNA [14].

Existing analysis on RNA-Seq data usually assumes Negative Binomial (NB) or the Log Normal (LN) distribution for RNA-Seq normalized counts *X* mapped to a gene [14, 16], with gene-wise dispersion summarized as a quadratic mean-variance relation *Var*(*X*) = *c* ∙ *E*(*X*)^2^. The square root of *c* coincides with coefficient of variation (CV) and depends on the assumed statistical distribution, i.e. $c=\phi +\frac{1}{\mu_1}$ for NB and *c* = exp(*σ*^2^) − 1 for LN [28], where *μ*_1_, *ϕ* are the mean and dispersion parameters of NB, and *σ* is the log standard deviation of LN, not related to log mean. Obviously, a drop in the gene-wise CV is expected to occur along with an increase in gene-wise mean, if the NB distribution assumption is valid for RNA-Seq counts. On the other hand, the gene-wise CV and mean should be independent if the assumed LN distribution is valid.

**Fig. 5:**
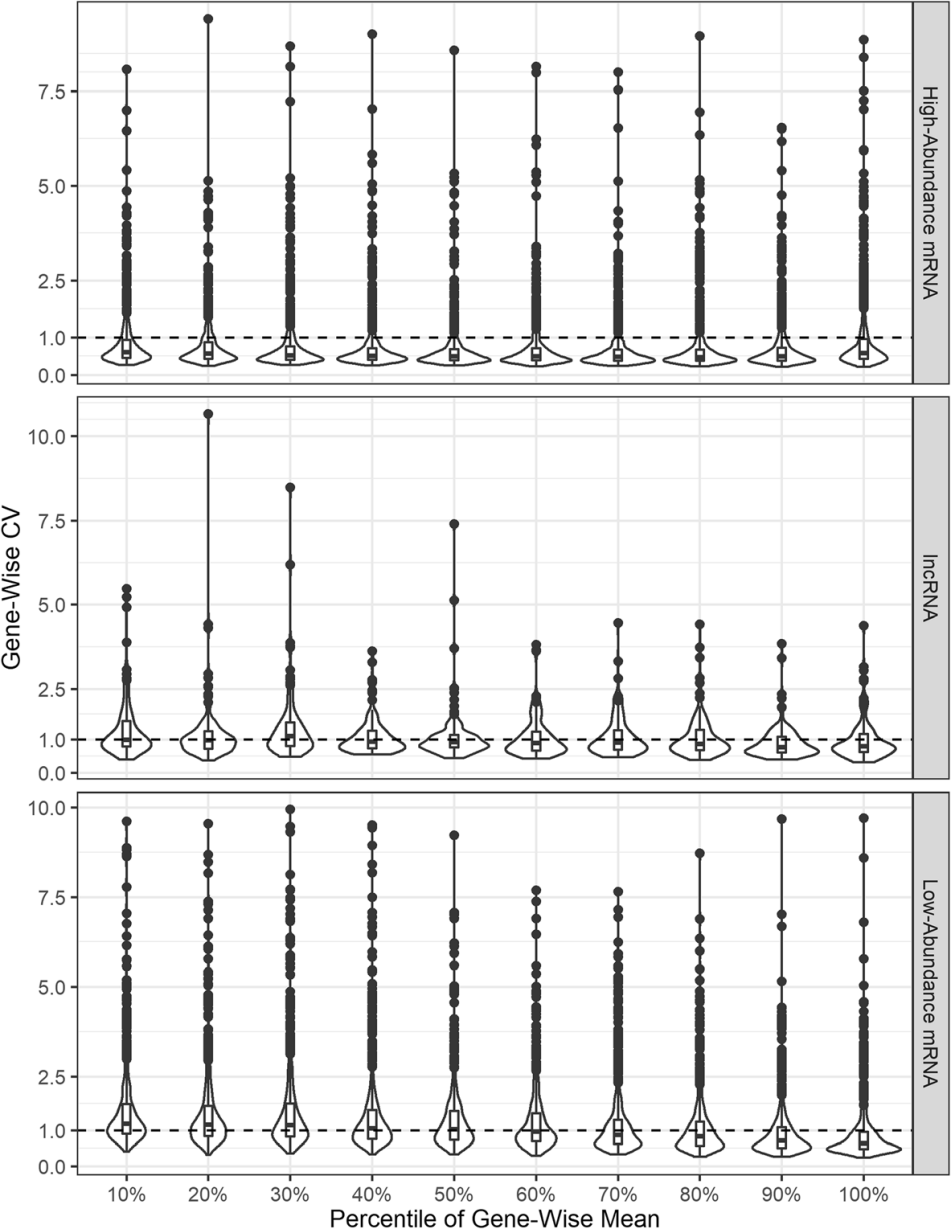
Gene-wise coefficient of variation. Violin and box plots for gene-wise coefficient of variation (CV) based on RPKM of three types of RNAs in TCGA HNSC study: high-abundance mRNA, lncRNA, and low-abundance mRNA. For each type of RNA, genes are divided into ten groups by the gene-wise mean percentiles

We first used the lncRNA and mRNA FPKM in the TCGA HNSC study [21] to investigate the dispersion patterns for three types of RNAs, i.e. high-abundance mRNA, low-abundance mRNA and lncRNA. Genes in lncRNA dataset were filtered by the criteria proposed by Yan et al. [9], while genes in mRNA dataset with more than 30% zero expression were removed. The cutoff between high vs. low abundant mRNA genes was the 85th percentile of gene-wise mean FPKM. We used the violin-box plots in Fig. 5 to illustrate the CV-mean relation for different RNAs in three panels. The totals of genes for each type of RNA are 9561 high-abundance mRNA genes, 8362 low-abundance mRNA genes, and 1322 lncRNA genes.

CVs for the majority of high-abundance mRNA genes were less than 1 and display a drop in higher expressed genes (Fig. 5). In contrast, the CV level for most of lncRNA and low-abundance mRNA genes in the lower two panels were close to CV = 1 and did not change along with gene-wise mean, especially for lncRNA genes with mean below the 80th percentile. The other genes in these panels severely deviated from CV = 1, and a negative CV-mean relation still existed in low-abundance mRNA when gene-wise mean increases from the 70th percentile to higher. We visualized and confirmed such CV-mean patterns via mRNA and lncRNA FPKM data in another two TCGA studies, i.e. Lung Squamous Cell Carcinoma (LUSC) and Lung Adenocarcinoma (LUAD), shown in Additional file 1: Figures S1-S2. We further assessed the dispersion patterns of mRNA low counts normalized by TMM and UQ methods [30, 31] (Additional File 1: Figure S3**)**. The similarity between different normalized mRNA counts implies that TMM or UQ normalized lncRNA counts also follow the CV-mean pattern of lncRNA RPKM in Fig. 5, although TMM and UQ normalized lncRNA counts in TCGA HNSC study were not publically available.

The expected CV level for lncRNA and low-abundance mRNA in Fig. 5 revealed an underlying statistical distribution in a large proportion of low abundant genes, which should have CV = 1 or *Var*(*X*) = *E*(*X*)^2^. This naturally leads to the Exponential distribution with density function $f(X)=\frac{1}{\lambda }{e}^{-\frac{X}{\lambda }}$, and *E*(*X*) = *λ*, *Var*(*X*) = *λ*^2^. In the light of fewer statistical parameters, it is of interest to consider the Exponential family as a latent distribution for low-counts RNA-Seq data, especially for lncRNA. We cannot ignore the fact that expression of certain lncRNA genes and low-abundance mRNA genes are still distributed as the well-known NB or LN family, illustrated by the genes with CV deviating from CV = 1 (Fig. 5 and Additional file 1: Figs. S1-S3). Therefore, in the presence of NB or LN-distributed counts, we adopted exponential family to account for the latent distribution of lncRNA genes and perform differential expression analysis.

### GLM with exponential likelihood

Let *Y*_*ij*_ be the lncRNA normalized counts for gene *i* in sample *j*, belonging to phenotype or treatment group *k*, *k* = 1, …, *K*. The generalized linear model (GLM) with the Exponential family is

$$ {Y}_{ij}\sim \mathrm{Exponential}\left({\lambda}_{ij}\right),{\lambda}_{ij}=E\left({Y}_{ij}\right) $$

Identity link: $$ {\lambda}_{ij}=\sum \limits_{k=1}^K{\beta}_{ik}{w}_{jk}+\sum \limits_{m=1}^M{\gamma}_m{v}_{jm.} $$

Logarithmic link*:* $$ \log \left({\lambda}_{ij}\right)=\sum \limits_{k=1}^K{\beta}_{ik}{w}_{jk}+\sum \limits_{m=1}^M{\gamma}_m{v}_{jm} $$

*w*_*jk*_ and *β*_*ik*_ are design matrix elements and coefficients for groups, while *v*_*jm*_ and *γ*_*m*_ (*m* = 1, …, *M*) are the *M* covariates and corresponding coefficients. Since *Y*_*ij*_ has been normalized for library size, this model does not include the RNA sequencing normalization factor, although it is a common parameter in existing tools based on NB assumption [28, 29, 32, 33].

In the absence of zero counts, lncDIFF uses the Exponential GLM for lncRNA DE analysis. Let *β*_*i*_ = (*β*_*i*1, …,_*β*_*iK*_) and *γ* = (*γ*_1, …,_*γ*_*m*_), for gene *i* with negligible zero occurrence (< 1%), the GLM likelihood based on the exponential density $f\left({Y}_{ij}\right)=\frac{1}{\lambda_{ij}}{e}^{-\frac{Y_{ij}}{\lambda_{ij}}}$ with identity or log link function is

1 $$ \mathrm{Identity}\ \mathrm{link}:L\left({\beta}_i,\gamma \right)={\sum}_{j=1}^Nl\left({\beta}_i,\gamma \right)={\sum}_{j=1}^N-\left[\frac{Y_{ij}}{\sum_{k=1}^K{\beta}_{ik}{w}_{jk}}+\log \left({\sum}_{k=1}^K{\beta}_{ik}{w}_{jk}+{\sum}_{m=1}^M{\gamma}_m{v}_{jm}\right)\right] $$

2 $$ \mathrm{Logarithmic}\ \mathrm{link}:L\left({\beta}_i,\gamma \right)={\sum}_{j=1}^Nl\left({\beta}_i,\gamma \right)={\sum}_{j=1}^N-\left[{Y}_{ij}{e}^{-\left(\sum \limits_{k=1}^K{\beta}_{ik}{w}_{jk}\right)}+{\sum}_{k=1}^K{\beta}_{ik}{w}_{jk}+{\sum}_{m=1}^M{\gamma}_m{v}_{jm}\right] $$

The exponential likelihood estimate for mean gene expression is the maximizer of *L*(*β*_*i*_, *γ*), that is $\left({\hat{\beta}}_i,\hat{\gamma}\right)= argmax\ L\left({\beta}_i,\gamma \right)$. Statistical models similar to Exponential GLM had been proposed and assessed in previous studies [34–37].

### Zero-inflated exponential likelihood

In lncRNA expression data, it is common to observe zero values in most genes at a non-negligible proportion (i.e., at least 1%) of samples. The excess zeroes and low counts for lncRNA cannot be addressed by integer models like Poisson and Negative Binomial (or Gamma-Poisson), especially for non-integer normalized counts in the range of (0, 2). Rounding decimals to integers and then applying Poisson or NB density [38, 39] or using data transformation, e.g. log2, voom, or VST [15, 16, 38] with limma [13, 40] may lead to errors in DE analysis. Therefore, we propose the zero-inflated quasi likelihood of Exponential GLM to account for the gene-wise inflation of zeros.

In order to incorporate the zero-inflated pattern, we re-write the normalized counts for gene *i* in sample *j* by a multiplicative error model [41–43] with random error *ϵ*_*ij*_, that is

3 $$ , {Y}_{ij}={\lambda}_{ij}{\epsilon}_{ij},E\left({\epsilon}_{ij}\right)=1 $$

The random errors *ϵ*_*ij*_ also have the occurrence of excess zeros with a prior probability mass *P*(*ϵ*_*ij*_ = 0) = 1 − π_*i*_, *P*(*ϵ*_*ij*_ > 0) = π_*i*_, and a continuous density at positive value with *E*(*ϵ*_*ij*_| *Y*_*ij*_ > 0) = γ, similar to [42, 44, 45]. If the non-zero expression *Y*_*ij*_ ∣ *Y*_*ij*_ > 0 follows an Exponential distribution (so does *ϵ*_*ij*_|*Y*_*ij*_ > 0), then the density functions for *Y*_*ij*_ including zero occurrence is

4 $$ f\left({Y}_{ij}\right)={\left(1-{\uppi}_i\right)}^{I_{\left({Y}_{ij}=0\right)}}{\left(\frac{\uppi^2}{\lambda_{ij}}{\mathrm{e}}^{-{\uppi}_i{Y}_{ij}/{\lambda}_{ij}}\right)}^{I_{\left({Y}_{ij}>0\right)}} $$

Equation (4) is derived in the Additional file 2. The corresponding likelihood function is

5 $$ {L}^{\ast}\left({\uppi}_i,{\beta}_i,\gamma \right)={\sum}_{j=1}^N{l_j}^{\ast}\left({\uppi}_i,{\beta}_i,\gamma \right) $$

*l*_*j*_^∗^(π_*i*_, *β*_*i*_, *γ*) is defined according to the selected link function as

6 $$ \mathrm{Identity}\ \mathrm{link}:{l_j}^{\ast}\left({\uppi}_i,{\beta}_i,\gamma \right)={I}_{\left({Y}_{ij}=0\right)}\log \left(1-{\uppi}_i\right)+{I}_{\left({Y}_{ij}>0\right)}\left(2\bullet \log \left({\uppi}_i\right)-\frac{\uppi_i{Y}_{ij}}{\sum_{k=1}^K{\beta}_{ik}{w}_{jk}}-\log \left({\sum}_{k=1}^K{\beta}_{ik}{w}_{jk}+{\sum}_{m=1}^M{\gamma}_m{v}_{jm}\right)\right) $$

7 $$ \mathrm{Logarithmic}\ \mathrm{link}:{l_j}^{\ast}\left({\uppi}_i,{\beta}_i,\gamma \right)={I}_{\left({Y}_{ij}=0\right)}\log \left(1-{\uppi}_i\right)+{I}_{\left({Y}_{ij}>0\right)}\left(2\bullet \log \left({\uppi}_i\right)-{\uppi}_i{Y}_{ij}{e}^{-\left(\sum \limits_{k=1}^K{\beta}_{ik}{w}_{jk}+\sum \limits_{m=1}^M{\gamma}_{ik}{v}_{jm}\right)}-\sum \limits_{k=1}^K{\beta}_{ik}{w}_{jk}-\sum \limits_{m=1}^M{\gamma}_m{v}_{jm}\right) $$

The zero-inflated maximum likelihood (ZI-ML) estimate for group-wise mean expression is the maximizer of *L*^∗^(π, *β*_*i*_, *γ*) in eq. (6), that is

8 $$ {\left({\hat{\uppi}}_{\boldsymbol{i}},{\hat{\beta}}_{\boldsymbol{i}},\hat{\gamma}\right)}_{ZI- ML}= argmax\ {L}^{\ast}\left({\uppi}_i,{\beta}_i,\gamma \right) $$

It is worthwhile to note that the likelihood function *L*^∗^(π_*i*_, *β*_*i*_, *γ*) in eq. (5) reduces to eqs. (1) and (2) if the proportion of zero expression is negligible, i.e. no more than 1%.

### Estimate group wise mean

For each gene, lncDIFF utilizes ${\left({\hat{\uppi}}_i,{\hat{\beta}}_i,\hat{\gamma}\right)}_{ZI- ML}$ in eq. (8) to estimate the mean expression level per group. We can prove mathematically that this estimate is asymptotically unbiased at large sample size, even though RNA-Seq low counts are usually a mixture of multiple distributions as previously reported [34–36]. Zero-inflated Poisson, NB, or LN likelihood may result in biased estimate for group wise mean gene expression in lncRNA low counts, due to limited mathematical power of these functions. Mathematical proof for unbiased estimate of group wise mean gene expression in lncDIFF is elaborated in the Additional File 2.

To illustrate the estimation accuracy of ${\left({\hat{\uppi}}_i,{\hat{\beta}}_i,\hat{\gamma}\right)}_{ZI- ML}$, we simply generated normalized lncRNA counts for a gene in three biological groups (i.e. groups A, B, C) without covariate effects by sampling from zero-inflated Exponential, NB, LN distributions, respectively. Each scenario contained 1000 replicates. The mean and median of 1000 estimated group effects were listed in Table 3, indicating that the presence of NB and LN-distributed low-counts did not have impact on the accuracy of group effect estimate in lncDIFF. In other words, lncRNA counts may occasionally deviate from Exponential family but does not affect the performance of lncDIFF. Hence, lncDIFF is a pseudo or quasi-likelihood [33] approach rather than a ‘true’ likelihood method for lncRNA low counts analysis.

**Table 1:** Top 20 significant genes from paired and unpaired lncDIFF analysis for TCGA HNSC study. The overlap of genes are in bold. Likelihood Ratio Test statistics, *p*-value and FDR are output from lncDIFF

Gene	Paired Tumor vs Normal		Statistics	FDR	Gene	Unpaired Tumor vs Normal		Statistics	FDR
	Ensembl ID	Log2 Fold Change				Ensembl ID	Log2 Fold Change		
***ERVH48–1***	**ENSG00000233056.1**	0.415	211.767	7.48E-45	***HCG22***	**ENSG00000228789.2**	−2.979	674.029	1.76E-145
***LINC02487***	**ENSG00000203688.4**	−3.747	200.441	1.11E-42	***LINC02487***	**ENSG00000203688.4**	−3.470	625.994	2.46E-135
***HCG22***	**ENSG00000228789.2**	−3.138	151.425	3.73E-32	*MYHAS*	ENSG00000272975.1	−0.935	324.216	7.70E-70
***LINC00668***	**ENSG00000265933.1**	2.189	148.534	1.20E-31	*LINC01405*	ENSG00000185847.3	−1.366	276.487	1.45E-59
***LINC02582***	**ENSG00000261780.2**	1.027	144.294	8.10E-31	*FALEC*	ENSG00000228126.1	− 1.721	252.647	1.82E-54
***LINC00941***	**ENSG00000235884.2**	2.450	138.020	1.59E-29	*TMEM238L*	ENSG00000263429.3	−2.250	235.559	8.06E-51
*LINC00942*	ENSG00000249628.2	1.105	128.195	1.92E-27	*AC005392.2*	ENSG00000231412.2	−2.342	198.936	6.73E-43
*LINC01234*	ENSG00000249550.2	1.755	121.173	5.79E-26	*AC140479.4*	ENSG00000261760.2	−1.471	188.314	1.23E-40
*LINC02154*	ENSG00000235385.1	2.099	120.529	7.12E-26	***ERVH48–1***	**ENSG00000233056.1**	0.444	185.507	4.47E-40
***AC134312.5***	**ENSG00000261327.3**	2.064	115.828	6.85E-25	*AC091563.1*	ENSG00000254343.2	−2.185	174.352	1.10E-37
***AL365181.2***	**ENSG00000272068.1**	1.191	111.605	5.24E-24	***LINC02582***	**ENSG00000261780.2**	1.009	161.008	8.19E-35
***DUXAP9***	**ENSG00000225210.5**	2.868	110.895	6.87E-24	***LINC00668***	**ENSG00000265933.1**	1.626	154.270	2.23E-33
***DUXAP8***	**ENSG00000206195.6**	2.422	105.798	8.30E-23	*ACBD3-AS1*	ENSG00000234478.1	−1.733	150.782	1.08E-32
*SFTA1P*	ENSG00000225383.2	1.676	103.239	2.80E-22	***LINC00941***	**ENSG00000235884.2**	2.090	150.692	1.08E-32
*AC010343.3*	ENSG00000250697.1	1.838	101.397	6.63E-22	***AC134312.5***	**ENSG00000261327.3**	2.146	150.725	1.08E-32
*ELFN1-AS1*	ENSG00000236081.1	1.590	101.238	6.74E-22	***DUXAP9***	**ENSG00000225210.5**	2.711	141.890	8.49E-31
*LINC00520*	ENSG00000258791.3	1.570	98.359	2.71E-21	*ABHD11*	ENSG00000225969.1	−1.730	140.148	1.92E-30
***AC134312.2***	**ENSG00000260162.2**	1.912	98.157	2.84E-21	***AL365181.2***	**ENSG00000272068.1**	1.008	138.932	3.35E-30
*AC114956.2*	ENSG00000248554.1	3.038	96.948	4.95E-21	***DUXAP8***	**ENSG00000206195.6**	2.230	134.501	2.95E-29
*CASC9*	ENSG00000249395.2	4.019	91.046	9.28E-20	***AC134312.2***	**ENSG00000260162.2**	1.982	129.028	4.42E-28

**Table 2:** lncDIFF group effect estimates and likelihood ratio test results of TCGA HNSC tumor vs. normal

Logarithmic link function					
Genes Ensembl ID	exp(*β*_*i*1_) (tumor)	exp(*β*_*i*2_) (contrast)	exp(*β*_*i*1_ + *β*_*i*2_) (normal)	*p*-value	FDR
ENSG00000005206.12	0.247	0.811	0.200	0.348	0.528
ENSG00000100181.17	0.737	0.993	0.732	0.974	0.982
ENSG00000126005.11	7.161	1.263	9.043	0.297	0.474
ENSG00000130600.11	181.885	1.571	285.661	0.044	0.115
ENSG00000131484.3	0.362	1.044	0.378	0.846	0.916
Identity link function					
Genes Ensembl ID	*β*_*i*1_ (tumor)	*β*_*i*2_ (contrast)	*β*_*i*1_ + *β*_*i*2_ (normal)	*p*-value	FDR
ENSG00000005206.12	0.247	−0.047	0.200	0.348	0.528
ENSG00000100181.17	0.737	−0.005	0.732	0.974	0.982
ENSG00000126005.11	7.160	1.887	9.047	0.297	0.474
ENSG00000130600.11	181.852	103.833	285.684	0.044	0.115
ENSG00000131484.3	0.362	0.016	0.378	0.846	0.916

**Table 3:** Estimated group effect on a gene by lncDIFF on simulated low-abundance expression. Low-abundance expressions were sampled from three statistical distributions and two scenarios of parameters (defined by *β* ’s and CV). 1000 replicates were generated resulting in 1000 estimates per scenario

Sampling Distribution	Groups	Baseline Group A	Contrast B vs A	Contrast C vs A	Baseline Group A	Contrast B vs A	Contrast C vs A
	True Parameter	*β*_*i*1_ = 2 (CV = 1.75)	*β*_*i*2_ = 3 (CV = 1.45)	*β*_*i*2_ = 8 (CV = 1.26)	*β*_*i*1_ = 2 (CV = 1)	*β*_*i*2_ = 3 (CV = 0.7)	*β*_*i*2_ = 8 (CV = 0.6)
Exponential (CV = 1)	Mean	1.98	3.03	7.91	1.98	3.03	7.91
	Median	1.97	3.02	7.85	1.97	3.02	7.85
Negative Binomial	Mean	1.99	3.00	8.03	2.00	3.01	8.06
	Median	1.98	2.96	7.97	2.00	3.01	8.02
Log Normal	Mean	1.99	2.99	7.98	1.99	3.00	8.02
	Median	1.95	2.92	7.82	1.97	2.95	7.95

### Detect differential expression by likelihood ratio test

For genes with non-Exponential low counts, the group wise mean expression level is independent of variance. Applying lncDIFF to these genes only detects the group effect on mean expression. On the other hand, declaring an Exponential-distributed low-counts gene as DE via lncDIFF implies significant group effect on both mean expression and variance, as log-mean is always half of log-variance in Exponential family. For differential analysis in lncDIFF, we apply the Likelihood Ratio Test (LRT) to the zero-inflated exponential likelihood function in eq. (5) to test hypothesis: *H*_0_ : *β*_*i*_ = *β*_*null*_ vs *H*_1_ : *β*_*i*_ = *β*_*full*_, where *β*_*null*_ is the design matrix coefficients with some equal to zero and *β*_*full*_ is the coefficients without zero.

The test statistic of LRT is *D* =  − 2*L*^∗^(*β*_*null*_) + 2*L*^∗^(*β*_*full*_) with *β*_*null*_ and *β*_*full*_ being the design matrix coefficients for null and alternative models. Let *m*_*null*_ and *m*_*full*_ be the number of distinct coefficients in *β*_*null*_ and *β*_*full*_. Test statistic *D* asymptotically follows *χ*^2^ distribution with degrees of freedom *m*_*full*_ − *m*_*null*_. The *p*-values from LRT are adjusted for multiple testing using the procedure of Benjamin and Hochberg false discovery rate [46]. The choice of link function does not affect the power of LRT, as illustrated by simulation study. An alternative algorithm to compute p-values for LRT is to use empirical distribution of LRT statistics *D* [39]. The empirical distribution of statistics *D* per gene can be generated by randomly shuffling the samples into *K* groups for *P* times and then calculate the LRT statistics for each permutation, that is *D*_1_, …, *D*_*P*_. Let the test statistics for the true groups be *D*_0_, then the empirical p-value is $\frac{\sum_{p=1}^P{I}_{\left({D}_p>{D}_0\right)}\ }{P}$, and can be adjusted by Benjamin and Hochberg procedure.

## Additional files

Additional file 1: **Figure S1.** Violin-box plots for gene-wise CV and mean for high, low-abundance mRNA and lncRNA FPKM in TCGA LUSC. **Figure S2.** Violin-box plots for gene-wise CV and mean for high, low-abundance mRNA and lncRNA FPKM in TCGA LUAD. **Figure S3.** Violin-box plots for gene-wise CV and mean for low-abundance mRNA counts normalized by TMM and UQ methods. **Figure S4.** Mean FDR for DE analysis on simulated data. Scenarios are in the order of gene-wise variance scale, from the smallest to the largest. **Figure S5.** Mean TPR for DE analysis on simulated data. Scenarios are in the order of gene-wise variance scale from the smallest to the largest. **Figure S6.** Survival time association with DE genes identified in both paired and unpaired TCGA HNSC tumor vs normal analysis. The 426 tumor samples are divided into two groups by the median of RPKM per gene. (A)-(D) are the Kaplan-Meier survival curves for genes *ERVH48–1*, *LINC00668, HCG22, LINC02582* individually*. (DOCX 2191 kb)*

Additional file 2: Supplementary Methods. (DOCX 22 kb)

Additional file 3: **Table S1.** mRNA genes highly correlated with each lncRNA DE gene for TCGA HNSC tumor vs normal. (XLS 26 kb)

## Data Availability

The public TCGA HNSC RNA-seq data was retrieved from: https://ibl.mdanderson.org/tanric/_design/basic/download.html IncDIFF source-code can be found at: https://github.com/qianli10000/lncDIFF

## Abbreviations

AUC: Area under the curve
CV: Coefficient of variation
DE: Differential expression
FDR: False discovery rate
FPR: False positive rate
GLM: Generalized linear model
HNSC: Head and neck squamous cell carcinomas
HPV: Human Papillomavirus
LFC: Log2 fold change
LN: Log Normal
lncRNA: Long non-coding RNA
LRT: Likelihood Ratio Test
NB: Negative Binomial
RPKM: Reads Per Kilobase per Million mapped reads
TCGA: The Cancer Genome Atlas
TPR: True positive rate
ZI-ML: Zero-inflated maximum likelihood
ZINB: Zero-inflated negative binomial
