# Supplementary material for: lncDIFF: a novel quasi-likelihood method for differential expression analysis of non-coding RNA
Source: BMC Genomics. 2019 Jul 2;20:539. doi: 10.1186/s12864-019-5926-4 (PMC6604377; doi:10.1186/s12864-019-5926-4)

**SUPPLEMENTAL FIGURES S1-S6**

**Figure S1:** Violin-box plots for gene-wise CV and mean for high, low-abundance mRNA and lncRNA FPKM in TCGA LUSC.

**
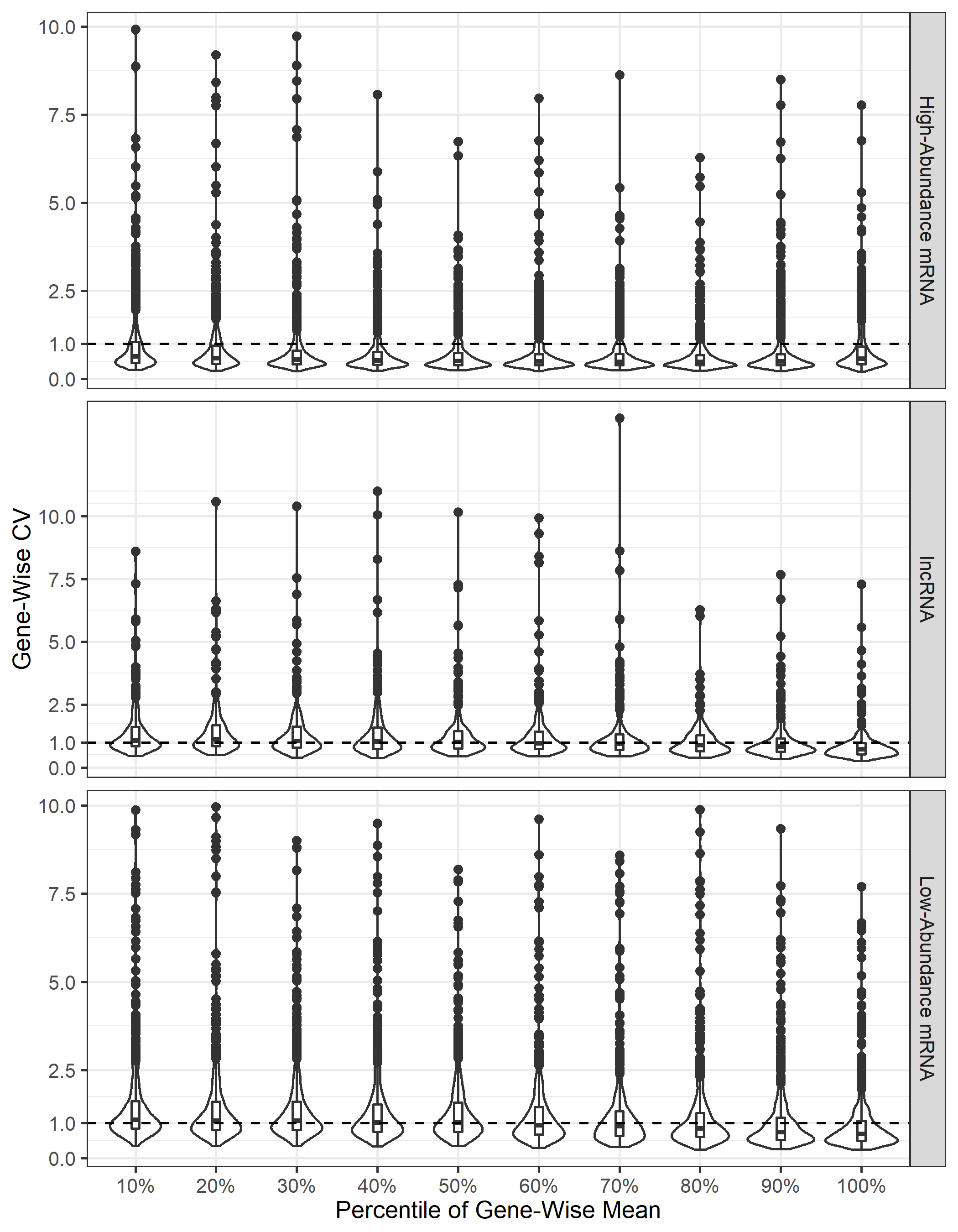
**

**Figure S2:** Violin-box plots for gene-wise CV and mean for high, low-abundance mRNA and lncRNA FPKM in TCGA LUAD.

**
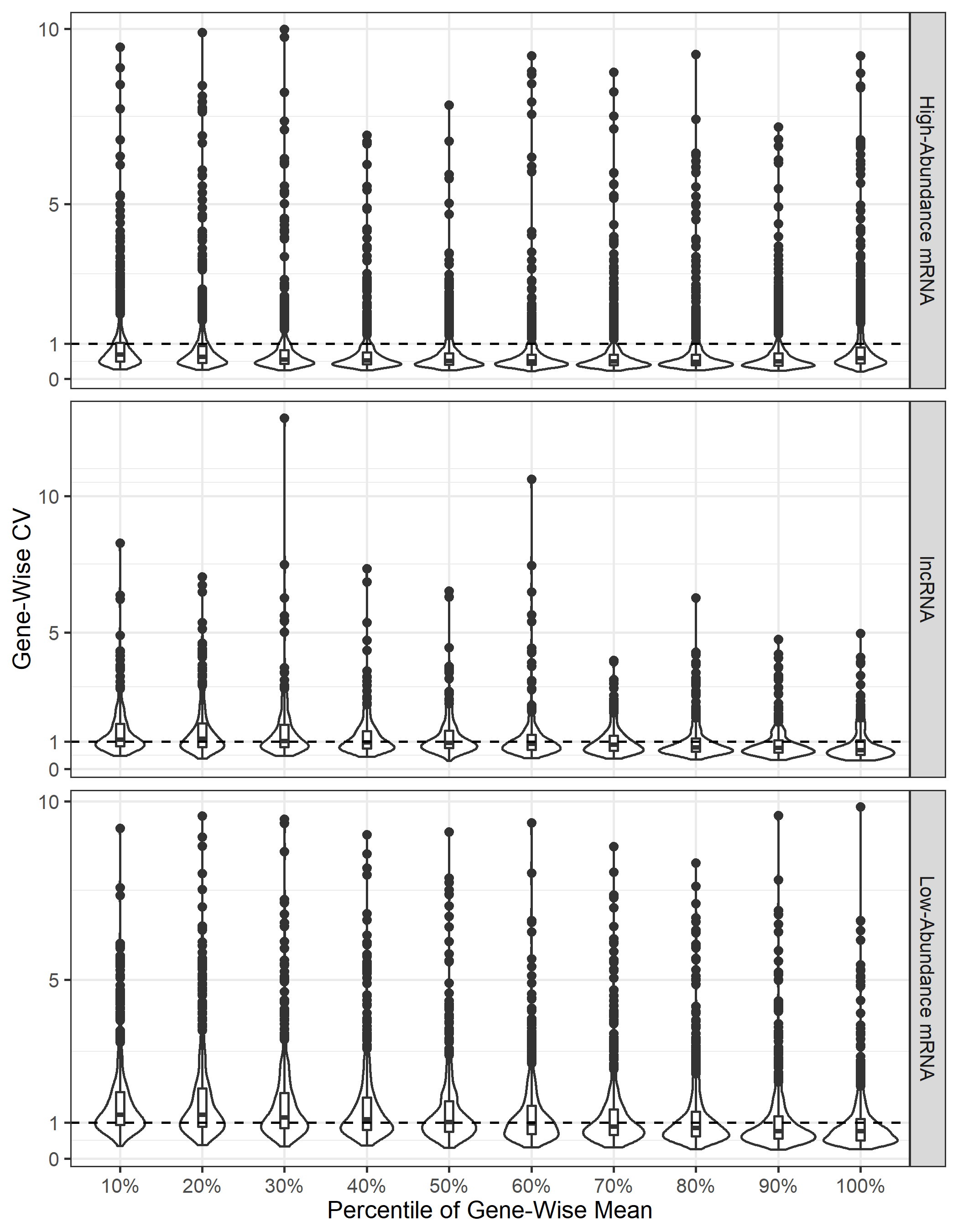
**

**Figure S3:** Violin-box plots for gene-wise CV and mean for low-abundance mRNA counts normalized by TMM and UQ methods.

**
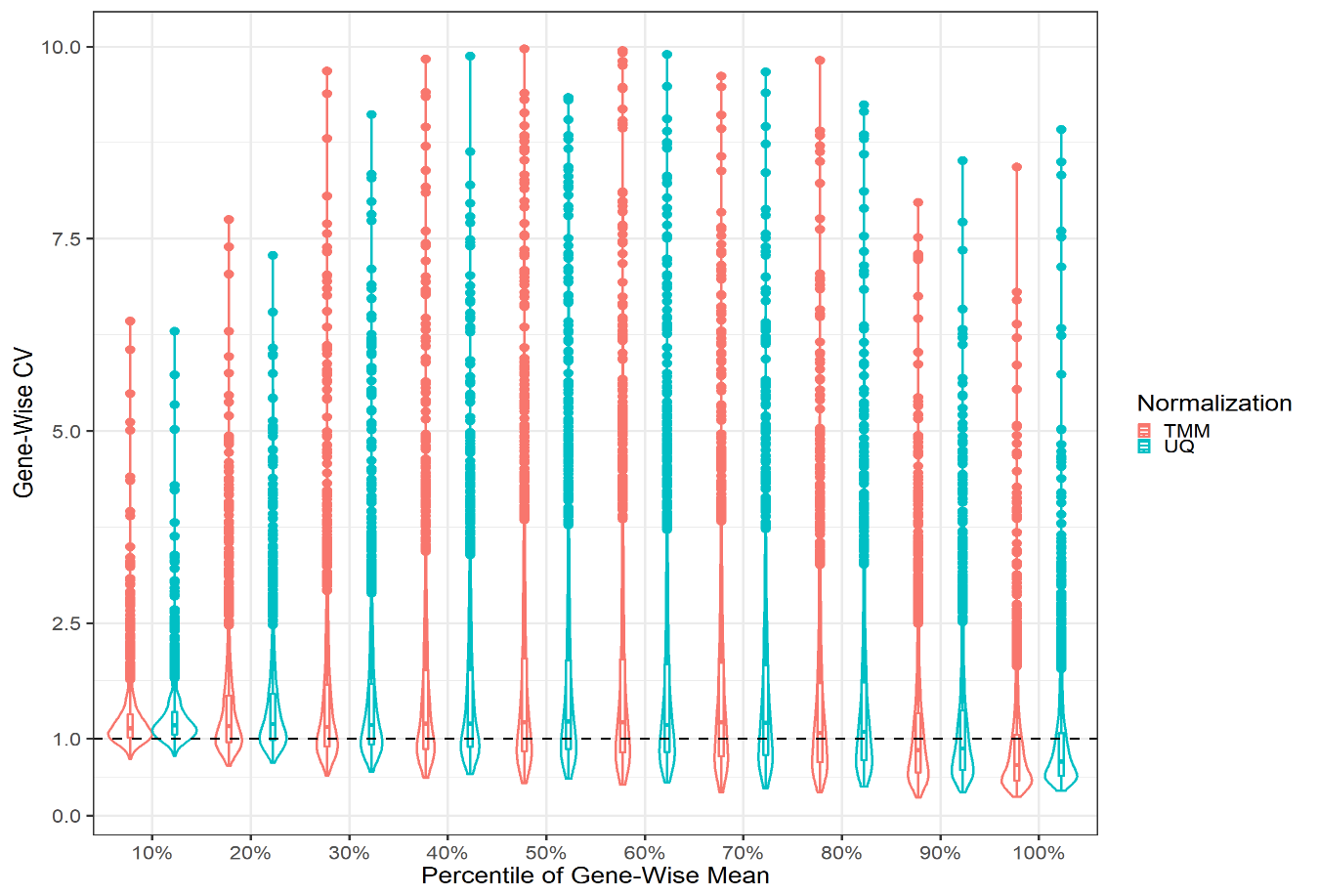
**

**Figure S4:** Mean FDR for DE analysis on simulated data. Scenarios are in the order of gene-wise variance scale, from the smallest to the largest.

**
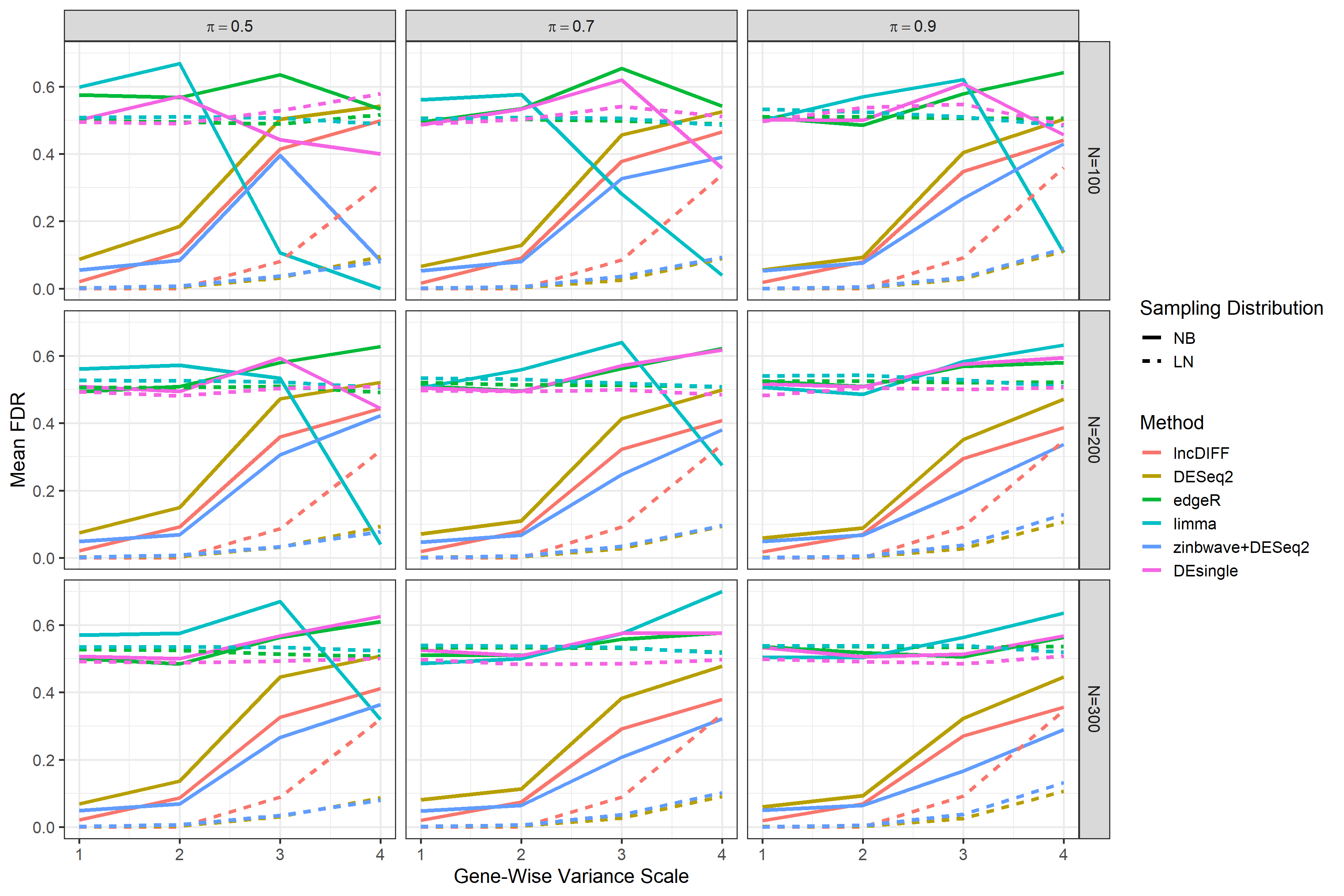
**

**Figure S5:** Mean TPR for DE analysis on simulated data. Scenarios are in the order of gene-wise variance scale from the smallest to the largest.


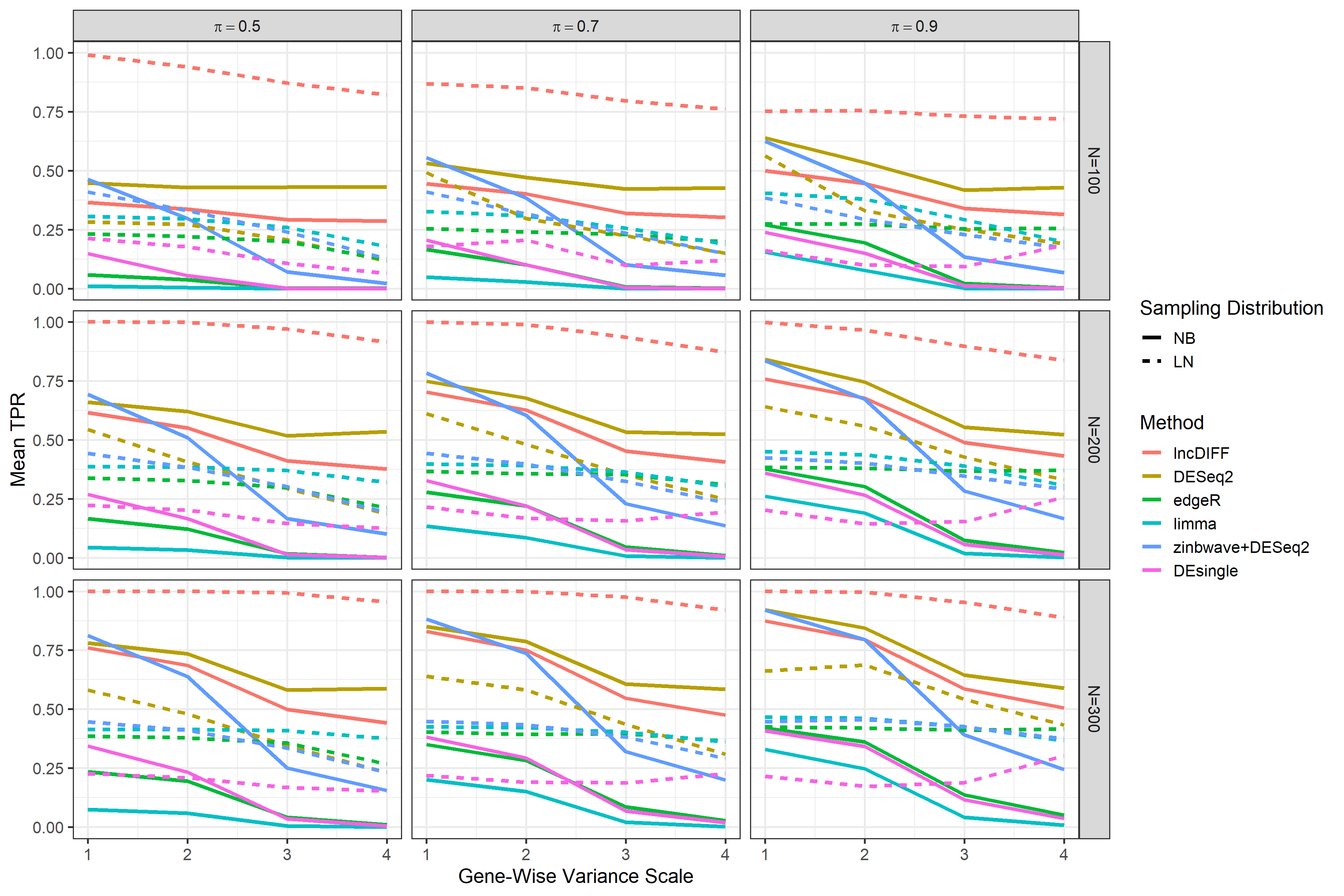


**Figure S6:** Survival time association with DE genes identified in both paired and unpaired TCGA HNSC tumor vs normal analysis. The 426 tumor samples are divided into two groups by the median of RPKM per gene. (A)-(D) are the Kaplan-Meier survival curves for genes *ERVH48-1*, *LINC00668, HCG22, LINC02582* individually*.*


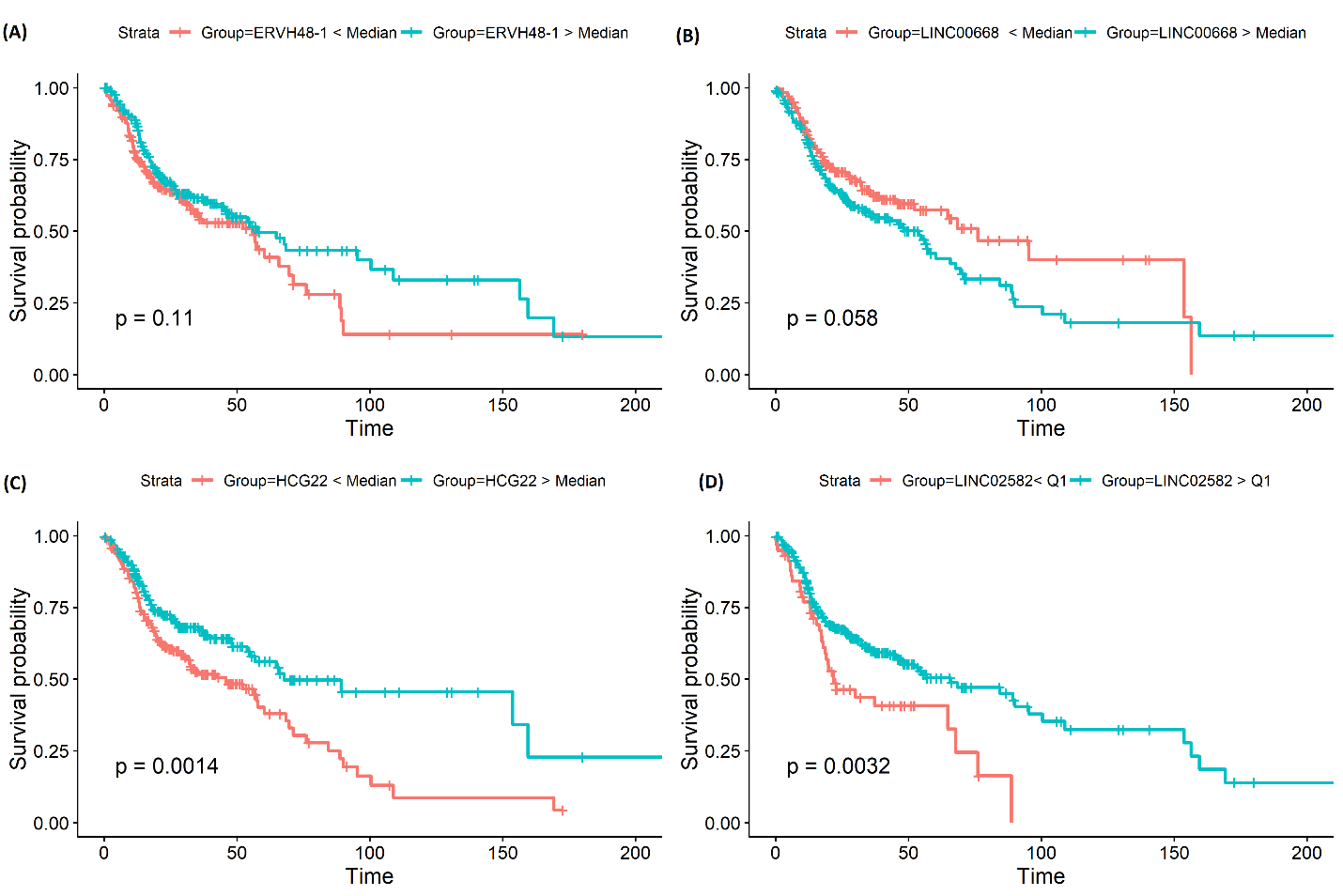

Supplement: Supplementary file 1 — Figure S1. Violin-box plots for gene-wise CV and mean for high, low-abundance mRNA and lncRNA FPKM in TCGA LUSC. Figure S2. Violin-box plots for gene-wise CV and mean for high, low-abundance mRNA and lncRNA FPKM in TCGA LUAD. Figure S3. Violin-box plots for gene-wise CV and mean for low-abundance mRNA counts normalized by TMM and UQ methods. Figure S4. Mean FDR for DE analysis on simulated data. Scenarios are in the order of gene-wise variance scale, from the smallest to the largest. Figure S5. Mean TPR for DE analysis on simulated data. Scenarios are in the order of gene-wise variance scale from the smallest to the largest. Figure S6. Survival time association with DE genes identified in both paired and unpaired TCGA HNSC tumor vs normal analysis. The 426 tumor samples are divided into two groups by the median of RPKM per gene. (A)-(D) are the Kaplan-Meier survival curves for genes ERVH48–1, LINC00668, HCG22, LINC02582 individually. (DOCX 2191 kb) [file 12864_2019_5926_MOESM1_ESM.docx]
