# Supplementary material for: lncDIFF: a novel quasi-likelihood method for differential expression analysis of non-coding RNA
Source: BMC Genomics. 2019 Jul 2;20:539. doi: 10.1186/s12864-019-5926-4 (PMC6604377; doi:10.1186/s12864-019-5926-4)
Supplement: Supplementary file 2 — Supplementary Methods. (DOCX 22 kb) [file 12864_2019_5926_MOESM2_ESM.docx]

**SUPPLEMENTAL METHODS**

1. **Zero-Inflated Exponential density for RPKM**$\boldsymbol{Y}_{\boldsymbol{ij}}$

For the multiplicative error model specified in equation (3), if the positive random error $\epsilon_{ij}|Y_{ij}>0$follows a distribution described by an Exponential density function$h\left( \epsilon_{ij}|Y_{ij}>0 \right)=\frac{1}{\gamma}e^{-\frac{\epsilon_{ij}}{\gamma}}$, then the distribution of $\epsilon_{ij}$ including zero occurrence is

$$g\left( \epsilon_{ij} \right)=\left( 1-\pi\right)^{I_{\left( \epsilon_{ij}=0 \right)}}\left( \frac{\pi}{\gamma}e^{-\frac{\epsilon_{ij}}{\gamma}} \right)^{I_{\left( \epsilon_{ij}>0 \right)}}$$

with${E(\epsilon}_{ij})=\pi\gamma$. According to the unit mean assumption ${E(\epsilon}_{ij})=1$ in equation (3), we have$\gamma=\frac{1}{\pi}$. That is,

$g(\epsilon_{ij})={(1-\pi)}^{I_{(\epsilon_{ij}=0)}}{(\pi^{2}e^{-\pi\epsilon_{ij}})}^{I_{(\epsilon_{ij}>0)}}$

Since $\epsilon_{ij}=Y_{ij}/\lambda_{ij}$, the semi-continuous distribution for $Y_{ij}$ can be derived by

$$f\left( Y_{ij} \right)= g(Y_{ij}/\lambda_{ij})\cdot\frac{d\epsilon_{ij}}{dY_{ij}}$$

That is,$f(Y_{ij})={(1-\pi)}^{I_{(Y_{ij}=0)}}{(\frac{\pi^{2}}{\lambda_{ij}}e^{-\pi Y_{ij}/\lambda_{ij}})}^{I_{(Y_{ij}>0)}}$.

1. **ZI-QML estimate** $\boldsymbol{(}\hat{\boldsymbol{\pi}}\boldsymbol{,}{{\hat{\boldsymbol{\beta}}}_{\boldsymbol{i}}\boldsymbol{,}\hat{\boldsymbol{\gamma}}\boldsymbol{)}}_{\boldsymbol{ZI-QML}}$ **is asymptotically unbiased.**

Proof: According to [1, 2], $(\hat{\pi}\boldsymbol{,}{\hat{\beta}_{\boldsymbol{i}}\boldsymbol{,}\hat{\gamma}\boldsymbol{)}}_{ZI-QML}$ is a consistent estimator if $L^{*}\left( \pi,\beta_{i},\gamma\right)$converges almost surely to${E[l_{j}}^{*}\left( {\pi,\beta}_{i},\gamma\right)]$ and ${E[l_{j}}^{*}\left( \pi,\beta_{i},\gamma\right)]$ is uniquely maximized at the true mean of RPKM, i.e.$\beta_{i\boldsymbol{0}}$. Suppose the true value of$\beta_{i}$,$\gamma$ are$\beta_{i\boldsymbol{0}}=\left( \beta_{i1\boldsymbol{0}},\ldots, \beta_{iK\boldsymbol{0}} \right),\gamma_{\boldsymbol{0}}=(\gamma_{1\boldsymbol{0},\ldots,}\gamma_{m\boldsymbol{0}})$ and the true value of $\pi$ is $\pi_{0}$.

1. Identity link function: $\lambda_{ij}=\sum_{k=1}^{K} \beta_{ik}w_{jk}+\sum_{m=1}^{M} \gamma_{m}v_{jm}$.

The true expectation of $Y_{ij}$ is$\lambda_{ij\boldsymbol{0}}=\sum_{k=1}^{K} \beta_{ik\boldsymbol{0}}w_{jk}+\sum_{m=1}^{M} \gamma_{m\boldsymbol{0}}v_{jm\boldsymbol{0}}$. Since $E\left( Y_{ij}|Y_{ij}>0 \right)=\lambda_{ij}/\pi$with true value$\lambda_{ij\boldsymbol{0}}/\pi_{0}$, it is not hard to show that ${E[l_{j}}^{*}\left( \pi,\beta_{i},\gamma\right)]$ is

${E[l_{j}}^{*}\left( \pi,\beta_{i},\gamma\right)] ={E[l_{j}}^{*}\left( \pi,\lambda_{ij} \right)]=\left( 1-\pi_{0} \right)\log\left( 1-\pi\right)+\pi_{0}(2\cdot log \left( \pi\right)-\frac{\pi\lambda_{ij\boldsymbol{0}}}{\pi_{0}\lambda_{ij}}-log(\lambda_{ij})) (7)$

which is a finite function. By law of large numbers, $L^{*}\left( \pi,\beta_{i},\gamma\right)$-the sample mean of ${l_{j}}^{*}\left( \pi,\beta_{i},\gamma\right)$-converges almost surely to ${E[l_{j}}^{*}\left( \pi,\beta_{i},\gamma\right)]$. Next, we need to demonstrate ${E[l_{j}}^{*}\left( \pi,\beta_{i},\gamma\right)]$ being uniquely maximized at ${(\pi_{0},\beta}_{i\boldsymbol{0}},\gamma_{\boldsymbol{0}})$.

We consider the maximizer of $A(\pi,\lambda_{ij})={E[l_{j}}^{*}\left( \pi,\lambda_{ij},\gamma\right)]+\pi_{0}log(\lambda_{ij\boldsymbol{0}})$ instead of${E[l_{j}}^{*}\left( \pi,\lambda_{ij} \right)]$.

That is

$${A\left( \pi,\lambda_{ij} \right)=E[l_{j}}^{*}\left( \pi,\lambda_{ij} \right)]=\left( 1-\pi_{0} \right)\log\left( 1-\pi\right)+\pi_{0}(2\cdot log \left( \pi\right)-\frac{\pi\lambda_{ij\boldsymbol{0}}}{\pi_{0}\lambda_{ij}}-log(\frac{\lambda_{ij}}{\lambda_{ij\boldsymbol{0}}}))$$

Let$x=\frac{\lambda_{ij}}{\lambda_{ij\boldsymbol{0}}}$, then

$$A\left( \pi,x \right)=A(\pi,\lambda_{ij})=\left( 1-\pi_{0} \right)\log\left( 1-\pi\right)+2\pi_{0}\log\left( \pi\right)-(\frac{\pi}{x}+\pi_{0}\log\left( x \right))$$

The first gradient of$A\left( \pi,x \right)$ is

$$\frac{\partial A\left( \pi,x \right)}{\partial\pi}=-\frac{1-\pi_{0}}{1-\pi}+\frac{{2\pi}_{0}}{\pi}-\frac{1}{x}=0 (8)$$

$$\frac{\partial A\left( \pi,x \right)}{\partial x}=\frac{\pi}{x^{2}}-\frac{\pi_{0}}{x}=0 (9)$$

The solution to equations (8) and (9) is $\left( \pi,x \right)=\left( \pi_{0},1 \right)$. The second gradient gives the Hessian matrix

$$H=\left[ \begin{matrix} \frac{\partial^{2}A\left( \pi,x \right)}{\partial\pi^{2}} & \frac{\partial^{2}A\left( \pi,x \right)}{\partial\pi\partial x} \\ \frac{\partial^{2}A\left( \pi,x \right)}{\partial\pi\partial x} & \frac{\partial^{2}A\left( \pi,x \right)}{\partial x^{2}} \end{matrix} \right]_{\left( \pi_{0},1 \right)}=\left[ \begin{matrix} \frac{1}{1-\pi_{0}}-\frac{2}{\pi_{0}} & 1 \\ 1 & -\pi_{0} \end{matrix} \right]$$

Since $\left| H \right|=\frac{1}{1+\pi_{0}}>0,$ $\left( \pi_{0},1 \right)$ is the unique maximizer of$A\left( \pi,x \right)$. Hence, $\left( \pi_{0},\lambda_{ij\boldsymbol{0}} \right)$ is the unique solution maximizing${E[l_{j}}^{*}\left( \pi,\lambda_{ij} \right)]$.

Lastly, we need to validate that $\lambda_{ij}=\lambda_{ij\boldsymbol{0}}$ implies ${(\beta}_{i},\gamma)=(\beta_{i\boldsymbol{0}}, \gamma_{\boldsymbol{0}})$. According to definitions, the design matrix, ${(\beta}_{i},\gamma)$ and $\lambda_{ij}$can be written as

$$\left[ \begin{matrix} w_{11} & \cdots& w_{1K} & v_{11} & \cdots& v_{1M} \\ \vdots& \cdots& \vdots& \vdots& \cdots& \vdots\\ w_{N1} & \cdots& w_{NK} & v_{N1} & \cdots& v_{NM} \end{matrix} \right]\left[ \begin{matrix} \begin{matrix} \beta_{i} \\ \gamma\end{matrix} \end{matrix} \right]=\left[ \begin{matrix} \lambda_{i1} \\ \vdots\\ \lambda_{iN} \end{matrix} \right] (10)$$

When $\lambda_{ij}=\lambda_{ij\boldsymbol{0}}$, equation (10) becomes

$$\left[ \begin{matrix} w_{11} & \cdots& w_{1K} & v_{11} & \cdots& v_{1M} \\ \vdots& \cdots& \vdots& \vdots& \cdots& \vdots\\ w_{N1} & \cdots& w_{NK} & v_{N1} & \cdots& v_{NM} \end{matrix} \right]\left[ \begin{matrix} \begin{matrix} \beta_{i} \\ \gamma\end{matrix} \end{matrix} \right]=\left[ \begin{matrix} \lambda_{i1\boldsymbol{0}} \\ \vdots\\ \lambda_{iN\boldsymbol{0}} \end{matrix} \right] (11)$$

and ${(\beta}_{i\boldsymbol{0}}, \gamma_{\boldsymbol{0}})$ is a solution to equation (11). Since the design matrix is of full rank, the solution to equation (11) is unique. Therefore, $\lambda_{ij}=\lambda_{ij\boldsymbol{0}}$ implies ${(\beta}_{i},\gamma)=(\beta_{i\boldsymbol{0}}, \gamma_{\boldsymbol{0}})$, and $(\pi_{0},\beta_{i\boldsymbol{0}}, \gamma_{\boldsymbol{0}})$ is the unique maximizer of ${E[l_{j}}^{*}\left( \pi,\beta_{i},\gamma\right)]$. The estimator $(\hat{\pi}, {\hat{\beta}_{i},\hat{\gamma})}_{ZI-QML}$ derived from ${l_{j}}^{*}\left( \pi,\beta_{i},\gamma\right)$ is asymptotically consistent.

1. Logarithmic link function:$log(\lambda_{ij})=\sum_{k=1}^{K} \beta_{ik}w_{jk}+\sum_{m=1}^{M} \gamma_{m}v_{jm}$.

The true expectation of $Y_{ij}$ is$\lambda_{ij\boldsymbol{0}}=e^{\sum_{k=1}^{K} \beta_{ik\boldsymbol{0}}w_{jk}+\sum_{m=1}^{M} \gamma_{m\boldsymbol{0}}v_{jm\boldsymbol{0}}}$. Similarly, we can derive ${E[l_{j}}^{*}\left( \pi,\lambda_{ij} \right)]$ as equation (7) and $L^{*}\left( \pi,\beta_{i} \right)$ converges almost surely to${E[l_{j}}^{*}\left( \pi,\lambda_{ij} \right)]$, which is uniquely maximized at $\left( \pi_{0},\lambda_{ij\boldsymbol{0}} \right)$. Similar to the above proof, ${E[l_{j}}^{*}\left( \pi,\beta_{ik^{'}},\gamma\right)]$ is uniquely maximized at$(\pi_{0},\beta_{i\boldsymbol{0}}, \gamma_{\boldsymbol{0}})$. Hence, consistency still holds for log link function.

**Reference**

1. Amemiya, T. and H.U. Press, *Advanced Econometrics*. 1985: Harvard University Press.

2. Gourieroux, C., A. Monfort, and A. Trognon, *Pseudo Maximum Likelihood Methods: Theory.* Econometrica, 1984. **52**(3): p. 681-700.
